# Efferent Auditory Pathways Inhibition in Turner syndrome

**DOI:** 10.1590/2317-1782/20212020300

**Published:** 2021-10-25

**Authors:** Martha Marcela de Matos Bazilio, Adriana Fernandes Duarte dos Santos, Silvana Frota, Marília Guimarães, Márcia Gonçalves Ribeiro

**Affiliations:** 1 Universidade Federal do Rio de Janeiro – UFRJ - Rio de Janeiro (RJ), Brasil.; 2 Instituto Nacional de Educação de Surdos – INES - Rio de Janeiro (RJ), Brasil.

**Keywords:** Turner Syndrome, XO Gonadal Dysgenesis, Efferent pathways, Superior olivary complex, Otoacoustic emissions

## Abstract

**Purpose:**

The goal of this study is to investigate the efferent auditory pathways inhibition in Turner's syndrome and to relate it to the cytogenetic profile.

**Methods:**

This is a cross-sectional study with a comparison group. A sample with 94 participants divided into two groups: The study group was a sample of 40 patients diagnosed with Turner’s syndrome (17.6 years of age). The control group was composed of 54 volunteers (18.9 years of age), female, without syndrome. The selected individuals were submitted to efferent auditory pathways inhibition research.

**Results:**

The mean of the inhibitory effect of the efferent auditory pathway in the study group in the right ear was 0.4 dB and in the comparison group it was 1.9 dB, however in the left ear the mean of the inhibitory effect of the efferent auditory pathway was 1.4 dB in the study group and 0.8 dB in the comparison group. The inhibitory effect of the efferent auditory pathway was present in 14 individuals with monosomy and in 15 with other cytogenetic alterations.

**Conclusions:**

In the study group, the efferent auditory pathways inhibition value was significantly higher in the left ear and significantly lower than the control group in the right ear. There was no significant difference in efferent auditory pathways inhibition of right ear and left ear between the karyotype types.

## INTRODUCTION

Turner syndrome (TS) is a chromosomal condition that occurs in approximately one out of every 2000 female live births ^(1)^. Monosomy of the X chromosome, structural changes, and mosaicism, which combines numerical and/or structural changes with a normal chromosomal lineage, in addition to the total or partial presence of the Y chromosome, are among the cytogenetic abnormalities classically associated with TS ^(1)^.

The main features in phenotypic females include proportional short stature, triangular face, retrognathism, high/ogival palate, neck webbing, low hair implantation, nipple hypertelorism, mild *pectus excavatum*, *cubitus valgus*, and sexual developmental deficiencies. There are renal and cardiovascular anomalies, in addition to impairment of visual and auditory functions ^(2)^. With respect to auditory aspects, several types of hearing loss related to TS have been commonly reported; however, few studies have associated them with central auditory aspects ^(1;3;4;5)^. Conductive hearing loss can be explained by the presence of ogival palate in TS patients, which fosters respiratory disorders and hinders secretion elimination, and thus may result in middle ear infections ^(3)^. Congenital change in the Eustachian tube anatomy is another causal factor to be considered ^(3)^. Progressive sensorineural hearing loss can also be observed in TS, and it can be explained by early presbycusis caused by estrogen deficiency in these patients ^(4;5)^. However, the pathophysiology involving the central auditory pathways in TS is still unknown ^(4)^. 

Hearing assessment involves not only peripheral hearing, but also the central auditory pathways since there are individuals who present hearing within the normal standards, but report hearing complaints, especially in noisy environments. Thus, it is important to know the integrity and functionality of the auditory system as a whole. The integrity of the afferent and efferent central auditory pathways, associated with their joint action, leads to the proper functioning of the central auditory system ^(6)^. The efferent auditory pathways act to modulate the cochlear outer hair cells, decrease the cochlear nerve action potential, protect against noise, locate the sound source, and improve detection of the sound source in noisy environments and selective attention ^(7;8)^. These abilities are fundamental to the correct processing of auditory information ^(9)^.

The integrity of the efferent auditory pathway, that is, the inhibitory effect of the efferent auditory pathway (IHEAP), can be assessed through attenuation of the otoacoustic emissions (OAE) in the presence of contralateral noise. This attenuation is a result of the action of the medial olivocochlear tract on the cochlear outer hair cells, reducing the gain in cochlear amplification and, consequently, the movement of the basilar membrane ^(10)^.

Among the features reported in TS, not only hearing loss is found, but also neurofunctional deficits ^(11)^ associated with sound localization, perception, and selective attention, which are functions attributed to the efferent auditory pathway ^(7,8)^. Knowledge about the auditory function in TS has been increasing and, to the best of our knowledge, the study of the condition of the efferent auditory pathway in that syndrome has not yet been addressed. This study aimed to investigate the IHEAP in TS patients and relate it to their cytogenetic profile.

## METHODS

This cross-sectional study with a comparison group was approved by the Research Ethics Committee of Instituto de Puericultura e Pediatria Martagão Gesteira and of Instituto Nacional de Educação de Surdos under the protocols 1864085 and 2960593, respectively. All study participants signed an Informed Consent Form (ICF) when needed. The hearing assessments were performed at Division of Audiology of the Instituto Nacional de Educação de Surdos by two trained and qualified audiologists.

The study sample was composed of 94 individuals aged 9-39 years old divided into two groups: study group (SG) and comparison group (CG). The SG was formed by a hospital-based convenience sample composed of 40 patients diagnosed with TS, with mean age of 17.6 years old (SD ±7.16), from genetic service of Instituto de Puericultura e Pediatria Martagão Gesteira and endocrinology service do Hospital Universitário Clementino Fraga Filho.

Individuals with cytogenetic diagnosis of TS verified by medical record of Cytogenetics Laboratory of The Instituto de Puericultura e Pediatria Martagão Gesteira (IPPMG) and/or Endocrinology Service of The Hospital Universitário Clementino Fraga Filho (HUCFF) were included in the SG. The exclusion criteria were as follows: the presence of any other genetic abnormality verified by the medical record; the presence of cognitive and/or neurological disorders that hindered the understanding of the instructions to conduct the examinations, observed through anamnesis; the presence of foreign body and/or wax, identified through ear examination; otological diseases of the outer ear (OE) and/or middle ear (ME), verified through immittance tests; hearing loss according to the World Health Organization (WHO) 1997 ^(12)^, that is, thresholds <25 dB at pure tone audiometry; speech recognition index (SRI) <88% ^(13)^, or absence of response to transient otoacoustic emissions (TOAE).

The CG was composed of 54 female volunteers, without TS, with mean age of 18.9 years old (SD ±7.1). Females in the age range similar to the individuals in the SG were included in the CG; the exclusion criteria were similar to those of the SG, in addition to not presenting short stature - a phenotype frequently found in TS.

After verification of the inclusion and exclusion criteria, two trained and qualified audiologists (MMMB and AFDS) carried out the inhibitory effect of the efferent auditory pathway (IHEAP) screening test through capturing and recording of TOAE with and without contralateral acoustic stimulus. The following parameters were used to assess the responses to the TOAE: stimulus intensity of 60 dB, reproducibility >70%, analysis window of ~20 ms, and frequency range of 1-5 KHz. The presence of response was considered when the amplitude of the TOAE was ≥3 dB SPL above the noise in at least three consecutive frequencies. This procedure was then repeated, but with the presence of a contralateral white noise at 60 dB SPL, that is, both the stimulus and the noise were applied at 60 dB (stimulus signal to contralateral noise ratio of 0 dB) ^(14)^. The TOAE were captured using an Eclipse EP25 device, whereas the noise was collected using an AD229B audiometer, both properly calibrated.

The IHEAP was determined by subtracting the response level of the TOAE without a contralateral white noise captured at a first moment (M1) from the response level of the TOAE with a contralateral white noise collected at a second moment (M2), as in the following formula ^(15)^:

IHEAP = TOAE without noise (M1) - TOAE with noise (M2)

*Source*: Modified form Collet, 1992 ^(15)^.

The responses were considered positive (presence of IHEAP) when there was a reduction ≥1 dB between the amplitude of the responses to the TOAE with and without the presence of a contralateral white noise, thus indicating normality of the efferent auditory pathway. The responses were considered negative (absence of IHEAP) when there was reduction <1 dB between the amplitude of the responses to the TOAE with and without presence of a contralateral white noise ^(15)^. 

After the IHEAP results of all participants were determined, the values of the SG were compared with those of the CG. The variations between moments M1 and M2 within each group were assessed using the Wilcoxon signed-rank test and the measures between the groups were compared using the Mann-Whitney test. Comparison of the categorical data (presence/absence of IHEAP) between groups was assessed by the chi-square (χ^2^) test.

The karyotypes found in the medical records were divided into two types: a) Type 1 (T1) - monosomy of the X chromosome and b) Type 2 (T2) - other cytogenetic abnormalities. Thus, the relation of the IHEAP result (present/absent) between the two karyotypes (T1 and T2) was verified and analyzed using the Fisher’s exact test. The analysis results were considered by the individual, not by ear; to this end, the IHEAP was considered present when at least one of the ears had a positive response.

Descriptive analysis presented the data in tables and illustrative graphs. The numerical data were expressed in measures of adequate central tendency and dispersion, whereas the categorical data were expressed as frequency and percentage.

In addition, a previous analysis was performed to verify the normality of the variables. To this end, the Shapiro-Wilk test was used together with a graphical analysis of the histograms. Once the variables presented non-Gaussian distribution in at least one moment and/or group, non-parametric tests were applied. Therefore, the most adequate measures for summarizing the data are quartiles (median and interquartile range [IQR: Q1-Q3]). The data were statistically processed using the SPSS 26 software. A significance level of 5% (*p*<0.05) was adopted for all statistical analyses.

## RESULTS

Initially, 88 patients with TS were assessed. Of these, 23 were excluded for presenting cognitive and/or neurological changes that hindered their understanding of the proposed tasks, one for having another associated syndrome, and 24 for showing peripheral auditory system disorders. Therefore, 40 individuals were included in the SG. As for the CG, all 54 female individuals assessed met the established inclusion criteria, and none of them were excluded from the study.

Table 1 shows the mean, median, and interquartile range (IQR: Q1–Q3) for the measures of the right (RE) and left (LE) ears at moments M1 and M2 according to groups (SG and CG).

**Table 1:** Descriptive measures of TOAE (RE and LE) at moments M1 and M2 and their respective IHEAP results according to the SG and CG

Variable	Study group (n = 40)						Comparison group (n = 54)						*p-value* 2
	mean	median	IQR				mean	median	IQR				
Right ear													
M1	18.2	19.0	15.3	-	23.0		18.2	19.0	14.0	-	23.0		0.94
M2	17.8	19.0	15.0	-	21.8		16.4	16.5	12.8	-	21.3		0.24
*p-*value^1^		0.87						0.001					
IHEAP	0.4	0	-1.8	-	1.0		1.9	1.0	-0.3	-	4.0		0.018
Left ear													
M1	18.4	18.0	16.3	-	21.8		17.9	19.0	13.0	-	22.3		0.90
M2	17.0	18.0	16.0	-	21.0		17.1	18.0	11.8	-	22.0		0.95
*p-*value*^1^*		0.014						0.089					
IHEAP	1.4	1.0	-0.8	-	2.8		0.8	1.0	-1.0	-	3.0		0.61

Data expressed as mean, median, and interquartile range (IQR: Q1-Q3)

1 Comparisons between moments M1 and M2 were evaluated using the Wilcoxon signed-rank test;

2 Comparisons between groups (study and comparison) were evaluated by the Mann-Whitney test

**Caption:** M1 = moment 1; M2 = moment 2; IQR = interquartile range; IHEAP = inhibitory effect of the efferent auditory pathway

Statistically significant variations (decrease) in the TOAE were observed from M1 to M2 for the LE (*p*=0.014) and RE (*p*=0.001) in the SG and CG, respectively. In addition, the SG showed a statistically significant decrease in the IHEAP response level on the RE compared with that of the CG (*p*=0.018).

**Figure 1:**
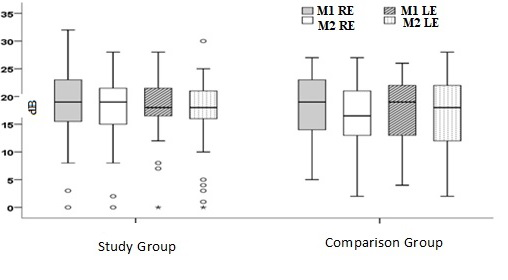
M1 = moment 1; M2 = moment 2; RE = right ear; LE = left ear; SG = study group; CG = comparison group; TOAE = transient otoacoustic emissions Figure 1. Medians of TOAE for the RE and LE at moments M1 and M2 (SG and CG)

Figure 1 shows the medians of the TOAE for the RE and LE at moments M1 and M2 according to group (SG and CG).

Table 2 shows the number (n) and percentage (%) values of the IHEAP for the RE and LE according to the SG and CG.

**Table 2:** Distribution of the IHEAP according to the SG and CG

IHEAP	Study group			Comparison group			*p-*value
	n	%		n	%		
Right ear							
Present	14	35.0		31	57.4		0.032
Absent	26	65.0		23	42.6		
Left ear							
Present	25	62.5		29	53.7		0.39
Absent	15	37.5		25	46.3		

Data expressed as number (n) and percentage (%) compared using the χ^2^ test.

**Caption:** IHEAP = inhibitory effect of the efferent auditory pathway

We observed that the presence of the IHEAP on the RE was significantly smaller in the SG than in the CG (*p*=0.032), and that there was no significant difference for the LE suppression between the groups.

Table 3 presents the number (n) and percentage (%) results of the IHEAP (present/absent) according to karyotype (T1 and T2).

**Table 3:** Distribution of the IHEAP according to karyotype (T1 and T2)

IHEAP	Present			Absent			*p-*value
	n	%		n	%		
Karyotype							
T1	14	48.3%		3	27.3%		0.20
T2	15	51.7%		8	72.7%		

Data expressed as number (n) and percentage (%) compared using the Fisher's exact test.

**Caption:** IHEAP = inhibitory effect of the efferent auditory pathway; T1 = type 1 = monosomy of the X chromosome; T2 = type 2 = other cytogenetic abnormalities

No significant difference in the IHEAP (present/absent) for the RE and LE was observed between the karyotypes.

## DISCUSSION

This study analyzed the inhibitory effect of the efferent auditory pathway (IHEAP) in individuals with Turner syndrome (TS). To the best of our knowledge, this is the first study addressing this measure in this group. The comparison group (CG) presented values significantly greater for the right ear (RE), and predominance of the response level on the left ear (LE) was observed in the study group (SG). This atypical condition suggests a possible cortical asymmetry related to the production and perception of speech and other neurocognitive abilities. In TS, neurocognitive disorders are varied, and difficulties are usually observed in non-verbal, visual-spatial/perceptual skills and memory, motor, executive and attentional functions ^(16)^. Although the central hemispheric predominance of one side over the other is a comprehensively studied topic, determining the different levels of this predominance for verbal and non-verbal sounds is a challenge, as there may be differences in asymmetry in the same individual ^(17)^.

On the other hand, it is known that the degree of mosaicism commonly varies between different types of tissues and organs. Even so, a recent study compared buccal mucosa smear samples on the right- and left-hand sides and observed a difference in the degree of mosaicism between the sides in half of the sample with TS, and the control showed no variation between the sides ^(18)^. This finding may be related to the data found in this study, which revealed that the IHEAP response was higher on the LE in participants with TS, considering that in addition to the knowledge about the possibility of all TS individuals present mosaicism ^(19)^, the tissues that constitute the central nervous system (CNS) and the buccal mucosa have the same embryological origin ^(20)^. The answer to the laterality found might be based on what was previously exposed.

Regarding the laterality of the IHEAP, that is, whether there is a difference in the response patterns of this measure between the right and left ears, there is an advantage of the RE, reinforcing the concept of laterality of the function of the olivocochlear system, which could indicate a delay in sound conduction in the left compared with the right olivocochlear tract ^(21)^. This finding can be observed in other studies that reported higher values and occurrence of the IHEAP on the RE in normal-hearing individuals ^(22)^. In addition, a study also verified an advantage of the RE over the LE only in the control group, and an advantage occurred in the LE in the study group ^(23)^. A study that compared pigmented and albino rats observed differences in the laterality of neurons in the efferent auditory system, providing evidence of a difference in the crossing pattern of the olivocochlear pathway in these animals ^(24)^.

There are controversies in the literature regarding the IHEAP, and these may be related to the variability of the method used in the studies, including the type of stimulus used (transient evoked or distortion product) - linear or non-linear, the signal-to-noise ratio considered, the use of raw or normalized index as a criterion for analyzing the results, and the differences between individuals within each sample. The present study applied the parameters most used in the literature ^(14)^: transient linear stimulus at an intensity of 60 dB, reproducibility >70%, analysis window of ~20 ms, and frequency range of 1-5 KHz. The presence of response was considered when the amplitude of the TOAE was ≥3 dB SPL above the noise in at least three consecutive frequencies. This procedure was then repeated, but with the presence of contralateral white noise at 60 dB SPL, and the raw criterion was used for analysis. The noise level associated with the level of response to the OAE is a relevant factor to quantify the IHEAP magnitude. Variations in the level of response to the OAE resulting from noise can be interpreted as physiological inhibition, that is, the low levels of signal-to-noise ratio of the OAE can hinder the interpretation of the IHEAP results ^(25)^. Despite the fact that the application protocol adopted a widely used (albeit weak) signal-to-noise ratio, this ratio of the OAE was >6 dB in the whole investigated sample (SG and CG), which is the minimum desirable level to detect a robust IHEAP and repeatable results ^(26)^. Moreover, there was no significant variation between the signal-to-noise ratio of the OAE in the SG and CG.

Even so, the findings of this study are in agreement with the literature, which reports that normal-hearing individuals may present IHEAP laterality on the RE. Nevertheless, it is relevant that future research comparing the IHEAP application and analysis criteria be conducted, so that such controversies can be minimized.

There is no consensus in the literature on the normality standard of the IHEAP ^(27)^, which makes it difficult to compare with previous studies. A study that compared smokers and non-smokers aged 20-31 years old used a normality standard of 0.5 dB to characterize the presence of the IHEAP ^(27)^. Another study that applied the IHEAP survey to adults of both genders aged 20-73 years old considered the presence of IHEAP response levels >0 dB ^(28)^. An IHEAP normality criterion ≥1 dB was adopted in this study because this is the value used in the vast majority of studies ^(14;15;26)^, as well as due to the characteristics of the equipment used. Low frequencies were observed regarding the IHEAP in this study both in the SG and CG (Table 2), which raises the hypothesis that the commonly used normality standard of 1 dB may be high, and suggests the need for further studies addressing the normality standard adopted for the presence of the IHEAP.

Research on the possible relation between the IHEAP and cytogenetic abnormality in TS did not show significant association in this studied sample, although a clinical tendency for presence of the IHEAP was observed in the T1 (with monosomy) group. Perhaps this significance can be verified in studies with larger sample sizes. As no studies addressing this theme in this population were found, data comparison was not possible.

Its sample size was a limitation to this study. Among the eligible individuals, 35.39% were excluded for presenting abnormal audiometry. Although this was not the objective of this study, we could observe that all participants with TS and normal audiometry (GS) presented responses to the TOAE (M1), which indicates normality of the cochlear function. This finding corroborates those of a study conducted with TS patients whose mean age was <40 years old ^(29)^. However, a study that evaluated 30 TS patients with a mean age of 52 years old found that 61.5% presented changes in the TOAE and only 46.2% had altered audiometry. This finding may be related to the fact that the mean age of the sample of the latter study was significantly higher than this study. Also, in the TOAE, there was no significant difference in the response level (M1) between the SG and CG for both ears. This finding could probably be different if the mean age of the sample of this study were higher since individuals with TS can present with early presbycusis ^(4)^ and, therefore, this possible cochlear change could impact the responses to the OAE. Because sensorineural hearing loss is commonly associated with TS ^(5;6)^, the application of the TOAE as a technique to monitor hearing in these patients could be used to detecting possible early cochlear lesions, considering that one of the functions of the OAE is monitoring the cochlear function, especially in patients at risk of hearing loss. This early identification can assist with the treatment, even before occurrence of changes in the audiometric thresholds ^(30)^.

It is worth emphasizing that this unprecedented study addressing the IHEAP in TS raises several questions concerning its occurrence, magnitude of responses, laterality, and association with the various TS cytogenetic abnormalities, demonstrating the need for further studies conducted with larger samples on this theme, so that more consistent conclusions can be reached.

## CONCLUSION

Values of the IHEAP were significantly greater for the LE and significantly smaller for the RE in the group of patients with Turner syndrome compared with those in the comparison group. No significant difference in the IHEAP of the RE and LE was observed between the karyotypes.
