# Supplementary material for: Efferent Auditory Pathways Inhibition in Turner syndrome
Source: Codas. 2021 Oct 25;34(1):e20200300. doi: 10.1590/2317-1782/20212020300 (PMC9886295; doi:10.1590/2317-1782/20212020300)
Supplement: Translation

# Translation: Efeito inibitório da via auditiva eferente na síndrome de Turner

**Authors:** Martha Marcela de Matos Bazilio, Adriana Fernandes Duarte dos Santos, Silvana Frota, Marília Guimarães, Márcia Gonçalves Ribeiro

**Affiliations:** 1 Universidade Federal do Rio de Janeiro – UFRJ - Rio de Janeiro (RJ), Brasil.; 2 Instituto Nacional de Educação de Surdos – INES - Rio de Janeiro (RJ), Brasil.

**Keywords:** Síndrome de Turner, Disgenesia Gonadal 45X, Via Eferente, Complexo Olivar Superior, Emissões Otoacústicas

## Abstract

**Objetivo:**

investigar o efeito inibitório da via auditiva eferente na síndrome de Turner e relacionar com o perfil citogenético.

**Método:**

estudo descritivo transversal com grupo de comparação. Amostra: Grupo estudo formado por 40 pacientes com síndrome de Turner (17,6 anos); e Grupo controle constituído por 54 indivíduos (18,9 anos), do sexo feminino sem síndrome. Os indivíduos selecionados foram submetidos à pesquisa do efeito inibitório da via auditiva eferente.

**Resultados:**

A média do Efeito inibitório da via auditiva eferente no grupo estudo na orelha direita foi 0,4 dB e no grupo comparação foi de 1,9 dB, entretanto na orelha esquerda a média do efeito inibitório da via auditiva eferente foi 1,4 dB no grupo estudo e 0,8 dB no grupo comparação. O efeito inibitório da via auditiva eferente foi presente em 14 indivíduos com monossomia e em 15 com outras alterações citogenéticas.

**Conclusão:**

No grupo estudo o valor do efeito inibitório da via auditiva eferente foi significantemente maior na orelha esquerda e significativamente menor que o grupo controle na direita. Não houve diferença significativa no efeito inibitório da via auditiva eferente entre os tipos de cariótipo.

## INTRODUÇÃO

A síndrome de Turner (ST) é uma alteração cromossômica que afeta aproximadamente um em cada 2000 nascidos do sexo feminino ^(1)^. Dentre as alterações citogenéticas relacionadas à síndrome temos classicamente a monossomia do cromossomo X, alterações estruturais e mosaicismo que conjuga alterações numéricas e/ou estruturais com linhagem cromossômica normal, além da presença total ou parcial do cromossomo Y ^(1)^.

O fenótipo característico da ST consta de baixa estatura proporcionada, face triangular, retrognatismo, palato ogival, pescoço curto e/ou alado, baixa implantação de cabelos na nuca, hipertelorismo mamilar, leve *pectus excavatum*, cúbito valgo e hipodesenvolvimento dos caracteres sexuais. Ocorrem anomalias renais e cardiovasculares, além de comprometimento da função visual e auditiva ^(2)^. Em relação aos aspectos auditivos, até o momento, comumente são relatados diversos tipos de perda auditiva na referida síndrome, porém poucos estudos fazem a associação com os aspectos auditivos centrais ^(1;3;4;5)^. A Perda auditiva condutiva pode ser explicadas pela presença do palato em ogiva nos pacientes com ST, o que facilita distúrbios respiratórios, e dificulta a eliminação de secreções, podendo resultar em infecções de orelha média ^(3)^. Outro fator causal a ser considerado são as mudanças congênitas na anatomia da tuba auditiva ^(3)^. Além disso, pode ser observado na ST perda auditiva neurossensorial progressiva, a qual pode ser justificada pela presbiacusia precoce ocasionada pela deficiência de estrogênio nesses pacientes ^(4;5)^. Entretanto, a fisiopatologia que envolve as vias auditivas centrais na ST ainda é desconhecida ^(4)^.

A avaliação auditiva engloba, não apenas a audição periférica, mas também as vias auditivas centrais, uma vez que existem pessoas que apresentam audição dentro dos padrões de normalidade, mas relatam queixa de não ouvir bem, principalmente em ambientes ruidosos. Desta forma, é importante o conhecimento da integridade e funcionalidade do sistema auditivo como um todo. A integridade das vias auditivas centrais aferentes e eferentes associada à ação conjunta das mesmas conduz ao funcionamento adequado do sistema auditivo central ^(6)^. As vias auditivas eferentes atuam na modulação das células ciliadas externas da cóclea, diminuição do potencial de ação do nervo coclear, proteção contra o ruído, localização da fonte sonora, melhora da detecção da fonte sonora em ambientes ruidosos e atenção seletiva ^(7;8)^. Estas habilidades são fundamentais para o correto processamento das informações auditivas ^(9)^.

A integridade da via auditiva eferente, ou seja, o efeito inibitório da via auditiva eferente (EIVE) pode ser avaliado através da atenuação das Emissões Otoacústicas (EOA) na presença de ruído contralateral. Esta atenuação ocorre devido à ação do trato olivococlear medial, nas células ciliadas externas, atenuando o ganho da amplificação coclear, e consequentemente, reduzindo a movimentação da membrana basilar ^(10)^.

Dentre as alterações relatadas na ST, podemos encontrar não apenas alterações auditivas, mas também déficits neuro funcionais ^(11)^ associados às funções de localização sonora, percepção e atenção seletiva, funções essas atribuídas à via auditiva eferente ^(7,8)^ . O conhecimento sobre a função auditiva na ST vem aumentando e até onde sabemos, o estudo da condição da via auditiva eferente na referida síndrome ainda não foi abordado. Este estudo teve como objetivo investigar o EIVE na ST e relacionar com o perfil citogenético.

## MÉTODO

Foi realizado a cross-sectional study com um grupo de comparação, aprovado pelo Comitê de Ética e Pesquisa do Instituo de Puericultura e Pediatria Martagão Gesteira (IPPMG/UFRJ) e do Instituto Nacional de Educação de Surdos (INES), sob os números: 1864085, 2960593 respectivamente. Todos os indivíduos que participaram do estudo assinaram o termo de consentimento livre e esclarecido e o termo de assentimento quando necessário. As avaliações auditivas foram realizadas na Divisão de Audiologia do Instituto Nacional de Educação de Surdos (DIAU/INES) por duas fonoaudiólogas capacitadas e treinadas.

Foram estudados 94 indivíduos com idade de 9 a 39 anos, que foram divididos em dois grupos: o Grupo estudo (GE) e o Grupo Controle (GC). O GE foi formado por amostra de conveniência de base hospitalar, composta por 40 pacientes diagnosticadas com ST, média de idade 17,6 anos (±7,16), procedentes dos Serviços de Genética Médica e de Endocrinologia Pediátrica do IPPMG/UFRJ e do Serviço de Endocrinologia do Hospital Universitário Clementino Fraga Filho (HUCFF/UFRJ).

Os critérios de inclusão para o GE foram: diagnóstico citogenético da ST, verificado através do registro no prontuário do Laboratório de Citogenética do IPPMG e/ou do serviço de Endocrinologia do HUCFF/UFRJ. Os critérios de exclusão foram: presença de qualquer outra alteração genética, verificada através do registro no prontuário; existência de alteração cognitiva e /ou neurológica que dificultasse a compreensão para as instruções de realização dos exames, observada através da anamnese; presença de corpo estranho e/ou rolha de cera, identificado através da meatoscopia; doenças otológicas de orelha externa (OE) e/ ou orelha média (OM), verificada através do exame de imitanciometria; perda auditiva, de acordo com a OMS 1997 ^(12)^,ou seja, limiares inferiores a 25 dB, na audiometria tonal; resultados no Índice de Reconhecimento de Fala (IRF) inferiores a 88% ^(13)^ ou ausência de respostas nas EOAT.

Já o GC foi constituído por 54 voluntárias, com média de idade 18,9 anos (±7,1), do sexo feminino sem ST. Os critérios de inclusão foram ser do sexo feminino em faixa etária semelhante ao do GE; os critérios de exclusão foram semelhantes ao do GE, somado ao fato de não apresentar baixa estatura, fenótipo frequente na ST.

Após a verificação dos critérios de inclusão e exclusão, duas fonoaudiólogas capacitadas e treinadas (MMMB e AFDS) aplicaram a avaliação do Efeito Inibitório da Via Eferente (EIVE), que foi avaliado através da captação e registro das EOAT com e sem estímulo acústico contralateral. Foram utilizados os seguintes parâmetros para realização das EOAT: estímulo à intensidade de 60 dB, reprodutibilidade maior que 70%, janela de análise de aproximadamente 20 ms, faixa de frequência de 1 KHz a 5 KHz. Foi considerada presença de resposta quando a amplitude das EOAT foi maior ou igual a 3 dB NPS acima do ruído, em no mínimo 3 frequências consecutivas. Este mesmo procedimento em seguida foi repetido, porém com a presença de ruído branco contralateral a 60 dB NPS, ou seja, tanto o estímulo como o ruído foram aplicados a 60 dB (relação sinal do estímulo e o ruído contralateral foi de 0 dB) ^(14)^. Para a captação das EOAT utilizamos o equipamento eclipse EP 25 e para o ruído utilizamos o audiômetro Ad 229-b, devidamente calibrados.

O EIVE foi determinado pela subtração do nível de resposta das EOAT sem ruído branco contralateral captado em um primeiro momento (M1) do nível de resposta das EAOT com ruído branco contralateral, captadas em um segundo momento (M2). A fórmula encontra-se a seguir ^(15)^. 

1.

EIVE = EOAT sem ruído (M1) - EOAT com ruído (M2)

Fonte: Modificado de Collet, 1992 ^(15)^

Foram consideradas respostas positivas (presença do EIVE) quando houve redução maior ou igual a 1dB entre amplitude de respostas das emissões otoacústicas sem e com presença de ruído branco contralateral, indicando normalidade da via auditiva eferente. Considerou-se ausência do EIVE, quando a redução da amplitude for inferior à 1dB, considerando a resposta das emissões com e sem presença de ruído branco contralateral (ausência do EIVE) ^(15)^. 

Após terem sido determinados os resultados do EIVE dos participantes de cada grupo, foram comparados os valores encontrados no GE com o GC. A variação entre os momentos M1 e M2 no interior de cada grupo foi avaliada pelo teste dos postos sinalizados de Wilcoxon e a comparação das medidas entre os dois grupos pelo teste de Mann-Whitney. A comparação entre os dados categóricos (presença/ausência do EIVE) entre os grupos foi analisada pelo *teste de qui-quadrado* (χ^2^).

Os cariótipos encontrados nos prontuários, foram divididos em dois tipos: a) o Tipo 1 (T1): monossomia do cromossomo X e b) Tipo 2 (T2): outras alterações citogenéticas. Sendo assim, verificamos a relação entre o resultado do EIVE (presente/ausente) entre os dois tipos de cariótipo (T1 e T2), que foi analisada pelo teste exato de Fisher. Foi considerada análise por indivíduo e não por orelha; para tal o EIVE foi considerado presente quando pelo menos uma das orelhas apresentou resultado positivo.

A análise descritiva apresentou os dados observados na forma de tabelas e gráficos ilustrativos. Os dados numéricos foram expressos pelas medidas de tendência central e de dispersão adequados, e os dados categóricos pela frequência e porcentagem.

Além disso, foi realizada uma análise prévia para verificar a normalidade das variáveis. Para tal, foi utilizado o teste de *Shapiro-Wilk* juntamente com a análise gráfica dos histogramas. Uma vez que as variáveis apresentaram distribuição não-Gaussiana, em pelo menos um momento e/ou grupo, foram utilizados testes não paramétricos. Sendo assim, as medidas mais adequadas para sumarização dos dados são pelos quartis (mediana e intervalo interquatílico (Q1-Q3). O critério de determinação de significância adotado foi o nível de 5%. A análise estatística foi processada pelo *software* estatístico SPSS versão 26.

## RESULTADOS

Inicialmente foram atendidas 88 pacientes com ST, sendo que 23 foram excluídas por apresentarem alteração cognitiva e/ou neurológica que dificultou a compreensão das tarefas propostas, uma por apresentar outra síndrome associada e 24 por apresentarem alteração auditiva no sistema auditivo periférico, sendo elegíveis 40 indivíduos para o estudo. Já para o GC, foram avaliados 54 indivíduos do sexo feminino e todas atenderam os critérios de inclusão estabelecidos e nenhuma foi excluída.

A Tabela 1 apresenta média, mediana e o intervalo interquartílico (IIQ: Q1 – Q3) das medidas da OD e OE nos momentos M1 e M2 segundo os grupos GE e GC.

**Tabela 1:** Medidas descritivas das EOAT (OD e OE) nos momentos M1 e M2 com os respectivos EIVE segundo os grupos GE e GC

Variável	Grupo Estudo (n = 40)						Grupo Controle (n = 54)						*p valor* 2
	média	mediana	IIQ				média	mediana	IIQ				
Orelha Direita													
M1	18,2	19,0	15,3	-	23,0		18,2	19,0	14,0	-	23,0		0,94
M2	17,8	19,0	15,0	-	21,8		16,4	16,5	12,8	-	21,3		0,24
*p valor* 1		0,87						0,001					
EIVE	0,4	0	-1,8	-	1,0		1,9	1,0	-0,3	-	4,0		0,018
Orelha Esquerda													
M1	18,4	18,0	16,3	-	21,8		17,9	19,0	13,0	-	22,3		0,90
M2	17,0	18,0	16,0	-	21,0		17,1	18,0	11,8	-	22,0		0,95
*p valor ^1^*		0,014						0,089					
EIVE	1,4	1,0	-0,8	-	2,8		0,8	1,0	-1,0	-	3,0		0,61

Os dados foram expressos pela média, mediana, e intervalo interquartílico IIQ: Q1-Q3.

1 A comparação entre os momentos M1 e M2 foi avaliada pelo teste dos postos sinalizados de Wilcoxon;

2 A comparação entre grupos (estudo e controle) foi avaliada pelo teste de Mann-Whitney

**Legenda:** M1 = momento 1; M2 = momento 2; IIQ = intervalo interquartílico; EIVE = Efeito Inibitório da via auditiva eferente

No grupo estudo, foi observado variação (queda) significativa do M1 para M2 na OE *(p-valor =0,014)* e no grupo controle foi observado queda significativa do M1 para M2 na OD *(p-valor = 0,001*). Além disso, verificou-se que o grupo estudo apresentou nível de resposta do EIVE significantemente menor na OD que o grupo *controle (p-valor = 0,018).*

A Figura 1 ilustra as medianas da OD e da OE nos momentos M1 e M2 segundo os grupos GE e GC.

**Figure 1:** M1 = momento 1; M2 = momento 2; OD = orelha direita; OE = orelha esquerda; GE = grupo estudo; GC = grupo controle; EOAT = Emissões Otoacústicas Transientes Figura 1. Medianas das EOAT das orelhas (OD e OE) nos momentos M1 e M2 (grupos GE e GC)

Na Tabela 2 é possível ver o número (n) e porcentagem (%) do EIVE da OD e OE segundo os grupos GE e GC.

**Tabela 2:** Distribuição do EIVE segundo os grupos GE e GC

EIVE	Grupo Estudo			Grupo Controle			*p valor*
	n	%		n	%		
Orelha Direita							
presente	14	35,0		31	57,4		0,032
ausente	26	65,0		23	42,6		
Orelha Esquerda							
presente	25	62,5		29	53,7		0,39
ausente	15	37,5		25	46,3		
							

Os dados foram expressos pela frequência (n) e porcentagem (%) e comparados pelo teste χ2

**Legenda:** EIVE = Efeito Inibitório da via auditiva eferente

Observou-se que o grupo estudo apresentou EIVE presente da OD significantemente menor que o grupo controle *(p-valor = 0,032),* e não houve diferença significativa na supressão da OE entre os dois grupos.

A Tabela 3 apresenta o número (n) e a porcentagem dos resultados do EIVE (presente / ausente) segundo o cariótipo (T1 e T2).

**Tabela 3:** Distribuição do EIVE segundo os tipos de cariótipo (T1 e T2)

EIVE	Presente			Ausente			*p valor*
	n	%		n	%		
Cariótipo							
T1	14	48,3%		3	27,3%		0,20
T2	15	51,7%		8	72,7%		

Os dados foram expressos pela frequência (n) e porcentagem (%) e comparados pelo teste de Teste exato de Fisher

**Legenda:** EIVE = Efeito Inibitório da via auditiva eferente; T1 = tipo 1 = monossomia do X; T2 = tipo 2 = outras alterações citogenéticas

Observou-se que não houve diferença significativa dos EIVE (presente/ausente) da OD e OE entre os dois grupos dos tipos de cariótipos.

## DISCUSSÃO

Este estudo analisou o Efeito inibitório da via auditiva eferente em indivíduos com a ST e até onde sabemos, este é o primeiro estudo sobre o EIVE na ST. O GC apresentou valores estatisticamente mais elevados na OD, e no GE houve predominância do nível de resposta na OE. Esta condição atípica nos leva a acreditar em uma possível assimetria cortical relacionada com a produção e percepção da fala e outras habilidades neurocognitivas. Na ST as alterações neurocognitivas são variadas, e geralmente, são observadas dificuldades em habilidades não verbais, visuo-espaciais/ perceptuais, memória, funções motoras, executivas e atencionais ^(16)^. Apesar da predominância hemisférica central de um lado sobre o outro ser um tema muito estudado, é um desafio determinar os diversos níveis de predominância hemisférica para os sons verbais e não verbais, pois pode existir diferença na assimetria em um mesmo indivíduo ^(17)^

Por outro lado, sabe-se que o grau de mosaicismo comumente varia entre os diferentes tipos de tecidos e órgãos, ainda assim em um estudo recente ao ser comparada a amostra de esfregaço bucal do lado direito e esquerdo, foi observada diferença no grau de mosaicismo entre os lados em metade da amostra avaliada com ST e o controle não apresentou variação entre os lados ^(18)^. Este achado pode estar relacionado com os dados encontrados em nosso estudo em que a resposta do EIVE foi mais elevada na OE dos participantes com ST, uma vez que além de saber da possibilidade de todas as pessoas com ST serem “mosaico” ^(19)^, os tecidos constituintes do SNC e da mucosa têm a mesma origem embriológica ^(20)^. Acreditamos que possa haver uma resposta para a lateralidade encontrada com base no que foi exposto acima.

Em relação à lateralidade do EIVE, ou seja, se existe diferença nos padrões de resposta do EIVE entre as orelhas direita e esquerda, sabe-se que existe vantagem da OD, o que reforça o conceito de lateralidade da função do sistema olivococlear, o que poderia indicar um atraso na condução sonora do trato olivococlear esquerdo em comparação com o direito ^(21)^. Este dado pode ser observado em outros estudos que apontam maiores valores e ocorrência de EIVE na OD em indivíduos normoouvintes ^(22)^. Além disso, outro estudo, também verificara a vantagem da orelha direita sobre a orelha esquerda apenas grupo controle e no grupo estudo a vantagem ocorreu na orelha esquerda ^(23)^. Em um estudo realizado, no qual comparou ratos pigmentados e albinos, observou-se diferenças na lateralidade dos neurônios do sistema auditivo eferente, fornecendo evidências de diferença no padrão de cruzamento da via olivococlear nesses animais ^(24)^. 

Há controvérsias na literatura sobre o EIVE, que pode estar relacionado com a variabilidade do método empregado nos estudos, como: tipo do estímulo utilizado (Transiente ou produto de distorção); linear ou não-linear; a relação sinal/ruído considerada, utilização do índice bruto ou normalizador como critério de análise do resultado e as próprias diferenças inter sujeitos dentro de cada amostra. Os parâmetros aplicados no presente estudo foram os mais utilizados na literatura ^(14)^ :estímulo Transiente, linear, à intensidade de 60 dB, reprodutibilidade maior que 70%, janela de análise de aproximadamente 20 ms, faixa de frequência de 1 KHz a 5 KHz. Foi considerada presença de resposta quando a amplitude das EOAT foi maior ou igual a 3 dB NPS acima do ruído, em no mínimo 3 frequências consecutivas. Este mesmo procedimento em seguida foi repetido, porém com a presença de ruído branco contralateral a 60 dB e utilizamos o índice bruto como critério de análise. O nível de ruído associado ao nível de resposta das EOA é um fator relevante para a quantificação da magnitude do EIVE. As variações no nível de resposta das EOA provenientes do ruído podem ser interpretadas como inibição fisiológica, ou seja, os baixos níveis de relação sinal/ruído das EOA pode dificultar a interpretação dos resultados do EVE ^(25)^. Apesar, de termos adotado em nosso protocolo de aplicação uma relação sinal ruído muito utilizada, porém considerada fraca, foi observada em toda a amostra estudada (GE e GC) relação sinal / ruído das EOA maior que 6 dB, sendo este o mínimo desejável para detectar EIVE robusto e resultados repetíveis ^(26)^. Além disso, não houve variação significativa entre a relação sinal/ruído das EOA do grupo estudo e do grupo controle.

Ainda assim, os dados encontrados concordam com a literatura em que indivíduos normoouvintes e sem alterações podem apresentar lateralidade do EIVE na OD, entretanto é relevante que pesquisas futuras sejam realizadas comparando os critérios de aplicação e análise do EIVE para que sejam minimizadas tais controvérsias.

Não existe consenso na literatura sobre o padrão de normalidade do EIVE ^(27)^, o que dificulta a comparação com estudos anteriores. No estudo onde foram comparados fumantes e não fumantes com idade entre 20 e 31 anos, o padrão de normalidade utilizado foi de 0,5 dB para caracterizar a presença do EIVE ^(27)^. Já em outro estudo, no qual foi aplicado a pesquisa do EIVE em adultos (20 a 73 anos) de ambos os sexos, considerou-se presença de EIVE níveis de resposta acima de 0 dB ^(28)^. No presente estudo adotamos como critério de normalidade EIVE maior ou igual a 1 dB por ser adotado em grande parte dos estudos ^(14;15;26)^, e devido às características do equipamento utilizado. Ao observarmos a ocorrência do EIVE, em nosso estudo, a frequência foi baixa tanto no GE quanto no GC (Tabela 2), levantando a hipótese que o padrão de normalidade de 1 dB comumente utilizado, possa ser elevado, sugerindo a necessidade de mais estudos sobre o padrão de normalidade adotado para o EIVE ser considerado presente.

Ao pesquisarmos a possível relação do EIVE com a alteração citogenética da ST, não encontramos associação significativa nessa amostra em estudo, embora no grupo T1 (grupo com monossomia) tenha sido observada uma tendência clínica a apresentarem EIVE presente. Talvez estudos com um tamanho amostral maior possam verificar tal significância. Comparação com estudos anteriores não pode ser realizada, pois não foram encontrados estudos realizados sobre o tema.

Uma limitação do estudo foi o tamanho amostral. Dentre os indivíduos elegíveis, 35,39% foram excluídos por apresentar audiometria alterada. Mesmo não sendo objetivo do nosso estudo, foi possível observar que todas as participantes com ST e audiometria normal (incluídas no GE) apresentaram resposta nas EOAT (M1), o que indica normalidade da função coclear. Tal achado é semelhante ao estudo realizado em pacientes com ST cuja média de idade foi inferior a 40 anos ^(29)^. Entretanto, um estudo que avaliou 30 pacientes com ST cuja média de idade foi de 52 anos, verificou que 61,5% apresentaram alteração das EOAT contra apenas 46,2% com alteração na audiometria. Este dado pode estar relacionado com a média de idade da amostra deste segundo estudo ser bem mais elevada do que o nosso. Ainda em relação às EOAT, observamos que não houve diferença significativa do nível de resposta (M1) entre o GE e o GC, tanto na OD quanto na OE. Esse achado provavelmente poderia diferir, se a média de idade da amostra do nosso estudo fosse maior, uma vez que indivíduos com ST podem apresentar presbiacusia precoce ^(4)^ e, portanto, essa possível alteração coclear poderia impactar nas respostas das EOA. Devido à perda auditiva neurosensorial ser comumente associada à ST ^(5;6)^, a aplicação das EOAT como técnica para monitoramento da audição desses pacientes, poderia ser utilizada com o objetivo de detectar possíveis lesões cocleares precocemente, pensando que uma das funções das EOA é o monitoramento da função coclear, principalmente em pacientes com risco de perda auditiva. Esta identificação precoce pode ajudar no tratamento, antes mesmo, que ocorra alteração nos limiares audiométricos. ^(30)^. 

É importante ressaltar que o estudo inédito sobre a via auditiva eferente na ST provoca vários questionamentos, seja em relação à sua ocorrência, magnitude de respostas, lateralidade e associação com as diversas alterações citogenéticas na ST, demonstrando a necessidade de pesquisas futuras sobre o tema, com maior quantidade de participantes para obtermos conclusões mais consistentes.

## CONCLUSÃO

No grupo de pacientes com ST o valor do EIVE foi significantemente maior na OE e significativamente menor que o grupo controle na OD. Não houve diferença significativa no EIVE da OD e da OE entre os dois tipos de cariótipo.
